# Application of a novel in vivo imaging approach to measure pulmonary vascular responses in mice

**DOI:** 10.14814/phy2.13875

**Published:** 2018-10-04

**Authors:** Melissa Preissner, Rhiannon P. Murrie, Catherine Bresee, Richard P. Carnibella, Andreas Fouras, E. Kenneth Weir, Stephen Dubsky, Isaac P. Pinar, Heather D. Jones

**Affiliations:** ^1^ Department of Mechanical and Aerospace Engineering Monash University Melbourne Victoria Australia; ^2^ Cedars‐Sinai Medical Center Biostatistics & Bioinformatics Research Institute Los Angeles California; ^3^ 4Dx Limited Melbourne Victoria Australia; ^4^ Department of Medicine University of Minnesota Minneapolis Minnesota; ^5^ Department of Biomedical Sciences Cedars‐Sinai Medical Center Biomedical Imaging Research Institute Los Angeles California

**Keywords:** 4DCT, in vivo imaging, micro‐CT, pulmonary vasculature

## Abstract

Noninvasive imaging of the murine pulmonary vasculature is challenging due to the small size of the animal, limits of resolution of the imaging technology, terminal nature of the procedure, or the need for intravenous contrast. We report the application of laboratory‐based high‐speed, high‐resolution x‐ray imaging, and image analysis to detect quantitative changes in the pulmonary vascular tree over time in the same animal without the need for intravenous contrast. Using this approach, we detected an increased number of vessels in the pulmonary vascular tree of animals after 30 min of recovery from a brief exposure to inspired gas with 10% oxygen plus 5% carbon dioxide (mean ± standard deviation: 2193 ± 382 at baseline vs. 6177 ± 1171 at 30 min of recovery; *P* < 0.0001). In a separate set of animals, we showed that the total pulmonary blood volume increased (*P* = 0.0412) while median vascular diameter decreased from 0.20 mm (IQR: 0.15‐0.28 mm) to 0.18 mm (IQR: 0.14‐0.26 mm; *P* = 0.0436) over the respiratory cycle from end‐expiration to end‐inspiration. These findings suggest that the noninvasive, nonintravenous contrast imaging approach reported here can detect dynamic responses of the murine pulmonary vasculature and may be a useful tool in studying these responses in models of disease.

## Introduction

In vivo imaging techniques to study the pulmonary vasculature in mice provide valuable information on pulmonary physiology and mechanisms of disease. For example, elegant experiments using intravital microscopy of the murine pulmonary microcirculation have provided novel insights into such processes as hypoxic pulmonary vasoconstriction (Tabuchi et al. 2008; Wang et al. 2012; Goldenberg et al. 2015), leukocyte migration (Ichimura et al. 2005), and endothelial calcium signaling (Kiefmann et al. 2009; Rowlands et al. 2011). Single‐photon emission computed tomography (SPECT) imaging can characterize lung perfusion patterns in mice (Jobse et al. 2012; Koba et al. 2013) but the low resolution of this technique is an inherent limitation. Resin instillation has also been used to generate a detailed radiopaque cast of the murine vasculature, which can then be imaged using high‐resolution micro‐CT scanning (Molthen et al. 2004; Faight et al. 2017), but this technique is a terminal procedure. To date, quantitative in vivo imaging of the murine pulmonary vascular tree has required both intravenous contrast and synchrotron‐based radiation (Sonobe et al. 2011; Porra et al. 2017).

Previously, this approach required synchrotron‐based x‐ray sources (Kitchen et al. 2005; Morgan et al. 2014). However, recent advances in compact imaging have made it possible to perform high resolution, high speed imaging in a laboratory (Tuohimaa et al. 2007; Pfeiffer et al. 2008; Bech et al. 2013; Zhou et al. 2013; Larsson et al. 2016; Preissner et al. 2018). These recent advances in preclinical imaging provide further steps toward development in the area of clinical applications (Bravin et al. 2013). A number of authors on this study used laboratory‐based 4DCT x‐ray imaging and postimage acquistion analysis (Samarage et al. 2016) to generate three‐dimensional, quantitative pulmonary vascular trees from scans acquired without intravenous contrast. We compared the measurements of vessel diameters obtained using this method to measurements obtained in the same animals via pulmonary angiography with intravenous contrast and found excellent correlation. Here, we describe the application of this technique to characterize the pulmonary vascular response over time in mice to exposure to hypoxic/hypercarbic gas exposure and recovery from such exposure, and the changes in pulmonary blood volume during inspiration with positive pressure mechanical ventilation. We noted dynamic changes in the number of vessels detected before and after brief hypoxic/hypercarbic gas exposure, which we hypothesize may reflect dilatation or constriction of vessels near the limit of detection. Furthermore, we demonstrate an increase in pulmonary blood volume but a decrease in median vessel diameter with inspiration during positive pressure ventilation. We propose that this novel imaging and image analysis approach will be a useful technique for studying in vivo pulmonary vascular responses in small animal models of lung disease.

## Methods

### Ethical approval and animals

Eight‐week old BALB/c female mice (*n* = 7) were obtained from the Monash Animal Research Platform (Monash University, Melbourne, VIC, Australia). All experiments were approved by the local Animal Ethics Committee of Monash University (Ethics Project MARP/2014/137; Melbourne, VIC, Australia) and conducted in accordance with the guidelines set out in the Australian Code of Practice for the Care and Use of Animals for Scientific Purposes. Authors of this manuscript understand the ethical principles under which the Journal of Physiology operates and our work complies with the animal ethics as outlined in the journal (Grundy 2015).

### Mechanical ventilation

Mice were anesthetized with intraperitoneal injections of a mix of ketamine (Parnell Australia Pty Ltd, Alexandria NSW, Australia) and xylazine (Xylazil‐20, Troy Laboratories Pty Ltd, Smithfield NSW, Australia) at doses of 150 mg/kg and 10 mg/kg respectively. The depth of anesthesia was determined to be adequate by the absence of a toe‐pinch reflex. During housing, prior to anesthesia, mice were provided with food and water ad libitum. Each mouse was orotracheally intubated and securely restrained in a custom‐built chassis (Dubsky et al. 2012) in a supine position. Mice were then ventilated using pressure control ventilation on a mouse ventilator (AccuVent, Notting Hill Devices, Melbourne, VIC, Australia). For the hypoxia/hypercarbia experiment (*n* = 4 mice, identified as M1 to M4), the ventilator settings were: inspiratory pressure of 18 cmH_2_0, 2 cmH_2_0 positive end‐expiratory pressure (PEEP), and inspiratory and expiratory times of 200 ms each (a respiratory rate of 150 breaths/min). Tidal volumes with these settings were 8.5 ± 2.1 *μ*L/gm (mL/kg). For the Expiration/Inspiration experiment (*n* = 3 mice, identified as M5 to M7), ventilator settings were: inspiratory pressure of 20 cmH_2_0, 2 cmH_2_0 PEEP, and inspiratory and expiratory times of 300 msec each (a respiratory rate of 100 breaths/min). Tidal volumes with these settings were calculated as 9.1 ± 0.8 *μ*L/gm (mL/kg). All tidal volumes were calculated from the CT images. Subcutaneous saline (500 *μ*L) was administered at the onset of mechanical ventilation (MV). Mice were kept warm using pocket warmers wrapped around the lower abdomen and legs. The end point of the study was euthanasia via cervical dislocation under anesthesia.

### Hypoxia/CO_2_ protocol

Mice were imaged at baseline on room air (“Baseline”), and then the inhaled gas for the ventilator was changed to 10% oxygen, 5% carbon dioxide, and 85% nitrogen balance. We used a gas with both decreased oxygen and increased carbon dioxide concentrations based on literature suggesting that increased inhaled carbon dioxide concentrations would enhance hypoxic vasoconstrictor effects on the pulmonary vasculature (Hyde et al. 1964; Noble et al. 1981; Orchard et al. 1983; Lumb and Slinger 2015). Inhaled oxygen percentage was measured using an oxygen controller (ProOX 110, BioSpherix, Parish, NY) to ensure inhaled oxygen had reached 10%, which takes approximately 5 min (Fig. 2) due to gas mixing within the ventilator lines, and then animals were imaged again immediately (“10%O_2_ + 5%CO_2_”), with a 10 sec delay while researchers moved from the x‐ray imaging room to the control room. It was not possible to wait for longer periods of hypoxic/CO_2_ gas exposure before imaging, because the animals became dyssynchronous with the ventilator within 8 min of exposure to hypoxic/CO_2_ gas (and 4DCT imaging scans required 5 min to obtain). Animals were then switched back to room air, and the inhaled oxygen concentration was monitored to ensure return to 21%. Animals were imaged again after 10 min (“10 m Post”) and 30 min (“30 m Post”) of ventilation with oxygen concentration greater than or equal to 19% (about 4 min after stopping hypoxic gas due to mixing within ventilator lines, Fig. 2). Arterial oxygen saturations were measured in mice before the Baseline and 10 m Postscans using a MouseOX pulse oximeter (STARR Life Sciences, Oakmont, PA). After imaging, oxygen saturations for one animal (M4) were monitored during re‐exposure to hypoxic/CO_2_ gas, and then on return to room air to generate the data for Figure 2.

### Imaging protocol

Imaging was conducted at the Laboratory for Dynamic Imaging, Monash University (Melbourne, VIC, Australia). The imaging for the hypoxia/CO_2_ experiment utilized a prototype small animal high‐speed, high‐resolution microfocus x‐ray imaging system (Kim et al. 2017; Preissner et al. 2018). For the Expiration/Inspiration (“Expir/Inspir”) experiment, a commercial version of the x‐ray imaging system was used (Notting Hill Devices, Melbourne, Australia). Both instruments utilized microfocus x‐ray sources and a high‐speed flat‐panel detector (PaxScan, Varian Medical Systems, Palo Alto, CA). Taking into account geometric magnification, images are captured at a frame rate of 30 Hz and an exposure time of 18 msec with an effective pixel size of 19 *μ*m. The mouse was positioned in the chassis in front of the x‐ray beam in the upright position. A high precision rotary stage (Zaber Technologies, Vancouver, Canada) was used to rotate the mice 360 degrees under mechanical ventilation for the four‐dimensional computed tomography (4DCT) scan. The imaging was synchronized with ventilation and gated to obtain 800 projection images of the lungs for CT reconstruction. A calibration scan of an acrylic cylinder with fiducials (Yang et al. 2006) was performed before and after the mouse scans. This process captures the tilt angle and center of rotation of the scan necessary for accurate CT reconstruction results. Mice were imaged four times for the hypoxia/CO_2_ experiment, and once for the Expiration/Inspiration experiment, and subsequently euthanized.

### Image analysis

Each 4DCT scan from all animals was phase‐binned and synchronized with the ventilator. CT analysis was performed using the peak inspiration CT reconstruction for the hypoxia/CO_2_ experiment, and using CT reconstructions from end‐expiration (“Expiration”) and end‐inspiration (“Inspiration”) phases of the respiratory cycle for the Expiration/Inspiration experiment.

The segmentation of the pulmonary vasculature was based on the contrast‐free pulmonary angiography (CFPA) technique by Samarage et al. (2016). This technique uses a Hessian‐based filter that uses multiple Gaussian scales to detect the probability of a tube‐like structure in the 3D image (Frangi et al. 1998). The algorithm defines a sharp threshold below which no vessels will be detected, which in our case, is in the range of 0.04–0.06 mm in diameter. The filtered image is used to obtain a 3D centerline tree of the segmented vessels with measurements of diameters mapped at equi‐distant points along the tree (“Measurements”). From this, various parameters, such as the distribution of diameters along the tree and the number of segments in the tree can be extracted. For the purposes of this study, we define a vessel as the segment between branching points along the centerline tree (“Vessels”). The segmentation was applied to the whole lung in order to capture the full range of visible pulmonary vessels, including the large conduit vessels.

### Pulmonary blood vessel volume calculation

The volume of the pulmonary blood vessels (outer diameter) for animals from the Expiration/Inspiration experiment (mice identified as M5 to M7) was calculated by obtaining a cylindrical volume for each segment in the centerline tree, i.e., by calculating the Euclidean distance between the points and using the branch (i.e., vessel) diameter obtained from the filtered image (described above) at each point. The individual segment volumes were then summed to obtain the aggregate volume for the lung, including the large conduit vessels.

### Tidal volume calculations

The tidal volumes for the hypoxia/CO_2_ mice (M1–M4) at baseline were calculated by converting the image to Hounsfield Units (HU) in order to obtain a quantitative measure of fraction of air per voxel, based on the standard definition of HU (Kalender 2005). The fractions were summed for the whole lung parenchyma and converted to volumes for both the end‐inspiration and the end‐expiratory images. The difference was calculated as the tidal volume (Fuld et al. 2008). The tidal volumes for the Expiration/Inspiration mice (M5, M6, M7) were estimated from the difference in the fraction of air in the lungs between inspiration and expiration in the CT images (Fuld et al. 2008).

### Heart rate measurements

Heart rates in the Expiration/Inspiration experiment (mice identified as M5 to M7) were measured using real‐time x‐ray videos of the thorax that were obtained during CT image acquisition (Videos S1‐S3). The number of heart beats for a 10 sec period was counted, and that number was multiplied by 6 to obtain beats per minute.

### Statistical analysis

Changes in the median vessel diameters were tested across animals over time using generalized linear mixed modeling techniques to adjust for the repeated measures within each animal and fitted to a gamma distribution given the skewed nature of the data. Average number of vessels and diameter measurements were tested across time with repeated measures ANOVA (hypoxia/CO_2_ model) or paired T‐test (Expir/Inspir model). Correlations between number of vessels and number of diameter measurements was constructed using mixed model linear regression to account for repeated measures across animals. Residuals were inspected to confirm the overall fit of all modeling. For all testing the level of significance was set at a two‐sided *P*‐value of <0.05 and post hoc testing was adjusted for multiple comparisons with Tukey T‐tests. Data are presented as means ± standard deviations (SD) or median ± interquartile range (IQR). Analysis was performed using SAS v9.4 software.

## Results

### Vascular tree reconstructions

**Figure 1:**
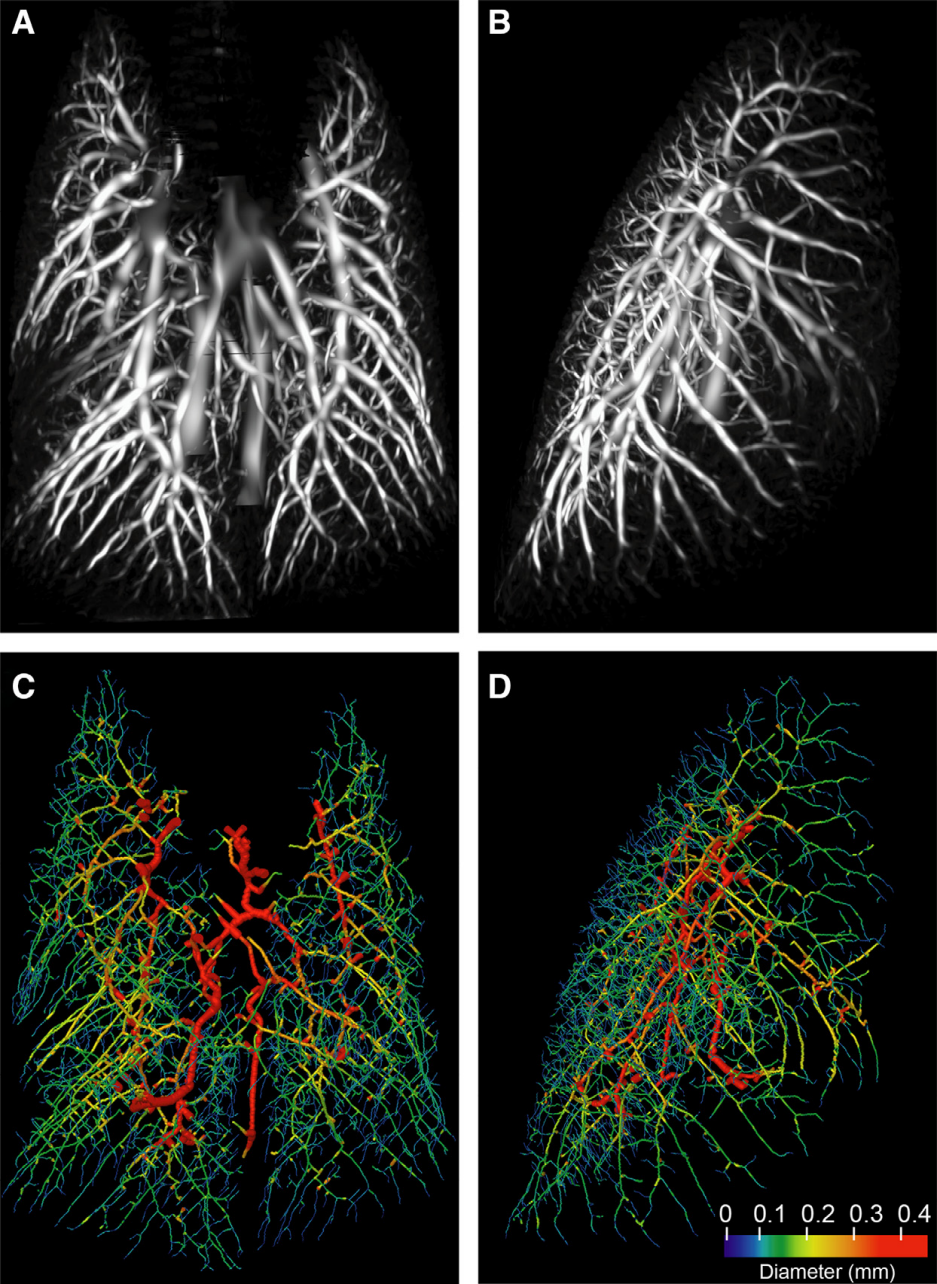
Murine pulmonary vasculature derived from a CT scan without intravenous contrast. A healthy 8 week old BALB/c mouse was scanned once using dynamic x‐ray imaging and end‐inspiration images were segmented as described under Methods. (A, B) Probability field of the filtered image, as a maximum intensity projection (A, frontal; B, lateral); (C, D) Vessel diameters mapped to the centerline tree for the same animal/scan (C, frontal; D, lateral). Branch thickness is reduced by a factor of 6.8 for clarity.

Reconstructed pulmonary vascular trees from the noncontrast CT scans were obtained using the CFPA image analysis technique developed and previously reported by some of the authors of this study (Samarage et al. 2016; Dubsky et al. 2017). Briefly, a filter is applied to the 3D CT image that detects tube‐like (i.e., vessel‐like) structures (Fig. 1) and provides a measurement of diameters at equi‐spaced points along the vessels (see Methods for details). Using this approach, a three‐dimensional vascular tree was generated for each scan (Dubsky et al. 2012). A range of 33,000 to 120,000 diameter measurements were obtained per vascular tree.

### Vessel diameter measurements during and after exposure to hypoxic/hypercarbic gas

**Figure 2:**
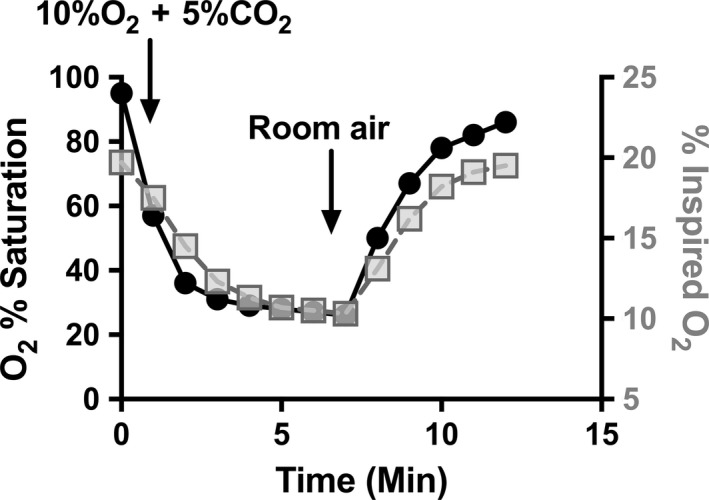
Changes in oxygen saturations in response to ventilation with hypoxic/CO_2_ gas. Noninvasive oxygen saturations (filled circles, left y axis) in one animal (M4) were tracked at baseline on room air, during ventilation with 10%O_2_ + 5%CO_2_, and during return to ventilation with room air after imaging was completed. Inspired oxygen concentration (grey squares, right y axis) was measured at the inspiratory limb of the ventilation circuit at the animal to determine rate of decline and recovery in actual inspired oxygen.

To test the hypothesis that hypoxic/hypercarbic pulmonary vasoconstriction could be detected using a noninvasive imaging approach, four sequential x‐ray 4DCT scans on each mouse were performed as follows: a baseline scan ventilating with room air (“Baseline”), a scan while ventilating with 10% oxygen and 5% carbon dioxide (“10%O_2_ + 5%CO_2_”), and then two more scans after 10 and 30 min of ventilation with room air posthypoxic/hypercarbic gas exposure (“10 m Post” and “30 m Post”). All animals had oxygen saturations of 93% or greater at baseline and upon return to room air ventilation. Ventilation with 10%O_2_ + 5%CO_2_ gas was confirmed to induce severe hypoxia within minutes by measurement of noninvasive oxygen saturations in one sample mouse (M4) after imaging and prior to euthanasia (Fig. 2).

**Figure 3:**
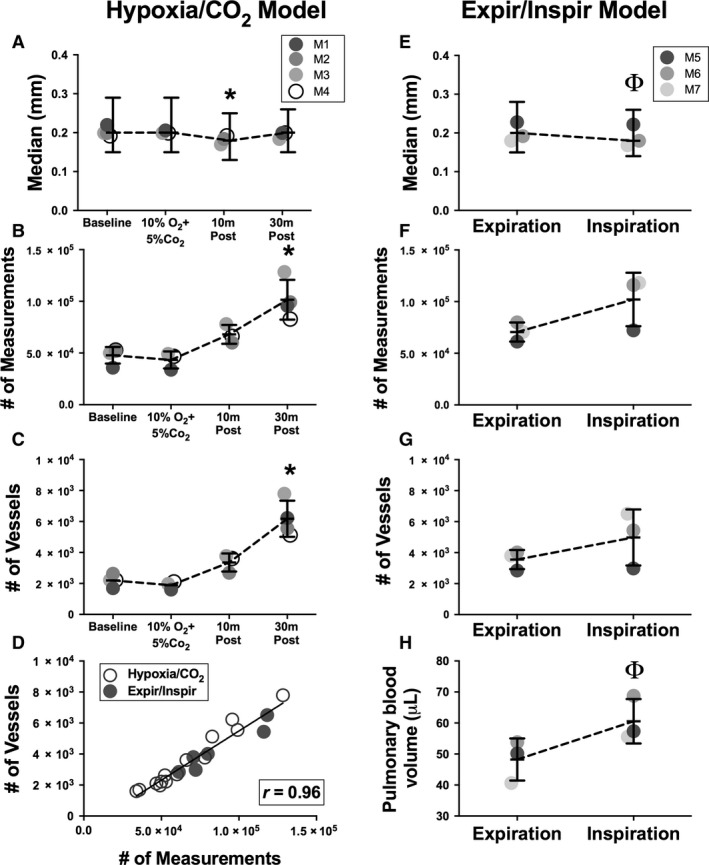
Measurements of changes in the murine pulmonary vascular tree during exposure to hypoxic/CO_2_ gas and with positive pressure mechanical ventilation. Three‐dimensional murine pulmonary vascular trees were reconstructed from CT scans performed without intravenous contrast. Median diameter measurements, numbers of diameter measurements and vessels, and pulmonary blood volumes were determined. (A, E) Median diameters (± IQR) of pulmonary vessels detected for individual mice in response to hypoxic/CO_2_ gas exposure (animals M1‐4) and from end‐expiration to end‐inspiration (animals M5‐7); (B, F) Mean ± SD number of diameter measurements that were able to be obtained from the vascular tree for each experiment; (C, G) Mean ± SD number of vessels that were detected from the vascular tree for each experiment; (D) Correlation of number of diameter measurements obtained to number of vessels detected from each vascular tree for all scans (*r* = 0.96, *P* < 0.0001); (H) Mean ± SD total pulmonary blood volume during positive pressure ventilation from end‐expiration to end‐inspiration. Baseline = no treatment; 10%O_2_ + 5%CO_2_ = during ventilation with 10% oxygen and 5% carbon dioxide; 10 m Post = 10 min of ventilation with room air after ventilation with 10%O_2_ + 5%CO_2_; 30 m Post = 30 min of ventilation with room air after ventilation with 10%O_2_ + 5%CO_2_. Expiration = data from vascular tree reconstructed from end‐expiratory phase of CT scan; Inspiration = data from vascular tree reconstructed from end‐inspiratory phase of same CT scan. *P < 0.001 in comparison to all other timepoints. (*P* < 0.05 in comparison to Expiration.

A three‐dimensional vascular tree for each scan was generated and vessel diameters were measured as described above. Two of 16 total scans (mouse M1 at 10 m Post and mouse M2 at 10%O_2_ + 5%CO_2_) were of insufficient quality to allow image analysis and so were excluded from this data set. At baseline, median vessel diameter was 0.20 mm (IQR: 0.15–0.29), which was unchanged during 10%O_2_ + 5%CO_2_ exposure (median 0.20; IQR: 0.15–0.29; *P* = 0.9347), or at 30 m posthypoxia/CO_2_ (median 0.20; IQR: 0.15–0.26; *P* = 0.9954). However, at the 10 m posthypoxia/CO_2_ time point, the median diameter was smallest at 0.18 mm (IQR: 0.13–0.25) which was significantly different from baseline (*P* = 0.0025), 10%O_2_ + 5%CO_2_ (*P* = 0.0095) and 30 m post (*P* = 0.0062); Fig. 3A, and Table 1).

**Table 1:** Vessel diameter data

Group	Timepoint	Number of diameter measurements (Mean ± SD)	Number of vessels detected (Mean ± SD)	Median vessel diameter (mm) (IQR)
Hypoxia/CO_2_ Model (*n* = 4 Mice)	Baseline	47,761 ± 8087	2193 ± 382	0.20 (0.15–0.29)
	10%O_2_+5%CO_2_	43,268 ± 8245	1890 ± 259	0.20 (0.15–0.29)
	10 m Post	68,065 ± 9172	3357 ± 583	0.18^a^ (0.13–0.25)
	30 m Post	101,508^a^ ± 19,257	6177^a^ ± 1171	0.20 (0.15–0.26)
Exp/Inspir Model (*n* = 3 Mice)	Expiration	70,613 ± 9305	3558 ± 623	0.20 (0.15–0.28)
	Inspiration	102,123 ± 25,995	4976 ± 1812	0.18^a^ (0.14–0.26)

Average number of diameter measurements, number of vessels detected, and median diameter measurements obtained from vascular trees for each animal across time points is shown.

a Indicates that data are significantly different than all other time points within group, *P* < 0.05.

Interestingly, the total number of diameter measurements acquired by the image analysis algorithm increased significantly at 30 min posthypoxia compared to all other time points (vs. baseline, *P* = 0.0024; vs. 10%O_2_ + 5%CO_2_, *P* = 0.0023; and vs. 10 m posthypoxia, *P* = 0.0419; Fig. 3B, Table 1). There are two possible explanations for this result from an image analysis perspective: (1) an increase in the length of vessels, which would lead the image analysis algorithm, which obtains diameter measurements of vessels at evenly spaced intervals, to generate more measurements per vessel; or (2) an increase in the total number of vessels detected, and therefore the number of vessels measured by the algorithm. For the purposes of this study, we defined a vessel as the segment between branching points along the centerline tree, and we assessed the number of individual vessels detected at each time point. The number of vessels detected increased and was significantly greater than all other time points at 30 min posthypoxia/CO_2_ (vs. baseline, *P* = 0.0005; vs. 10%O_2_ + 5%CO_2_, *P* = 0.0005; and vs. 10 m posthypoxia/CO_2_, *P* = 0.0063; Fig. 3C, Table 1). Using the data from all scans in this report (i.e. both the hypoxia/CO_2_ and Expiration/Inspiration experiments), we confirmed that the number of blood vessels imaged has a direct correlation with the number of diameter measurements acquired for each scan (Fig. 3D; Pearson's correlation: *r* = 0.9645, *P* < 0.0001).

**Figure 4:**
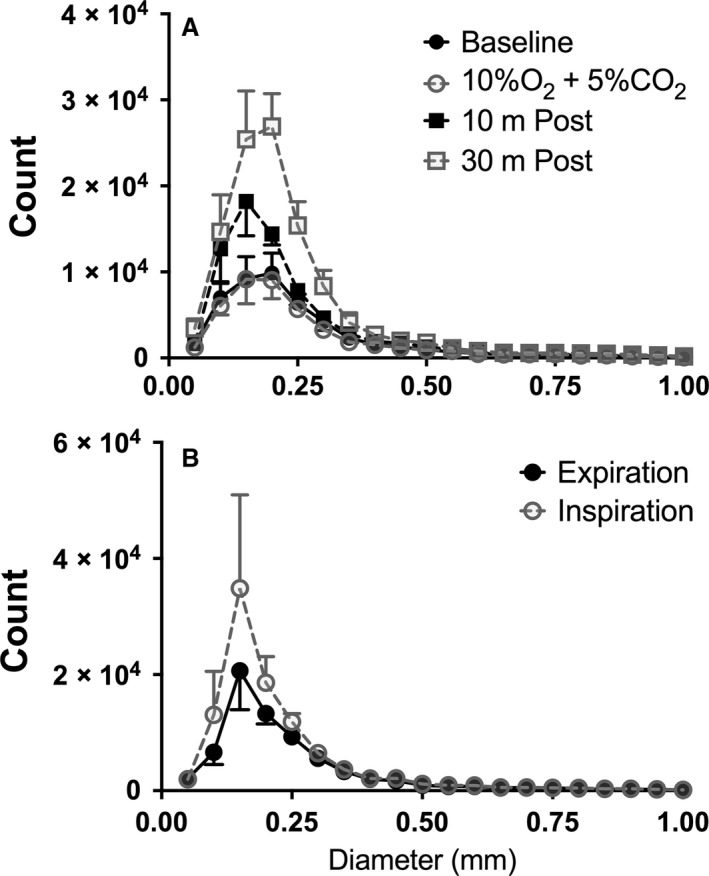
Distribution of diameter measurements from murine pulmonary vascular trees during exposure to hypoxia/hypercarbia (A) or from expiration to inspiration with positive pressure ventilation (B). Histogram of data counts are presented as the mean (± SD) across animals per 0.05 mm unit range of detected vessel diameters. The peak (mode) number of diameter measurements are at 0.16 mm for hypoxia/CO_2_ data and 0.17 mm for Expir/Inspir data.

We examined the distribution of diameter measurements in the hypoxia/CO_2_ experiment for all mice over all time points. The peak number of diameter measurements, that is, the mode, was the same in all scans and occurred at a diameter of 0.16 mm (Fig. 4A).

### Pulmonary blood volume increases during positive pressure inspiration

We were interested in understanding how vascular volumes and diameters change over the respiratory cycle during positive pressure ventilation and whether noninvasive imaging and image analysis techniques could provide insight into this phenomenon. We scanned three mice and reconstructed the three‐dimensional vascular tree from each animal using the CT scan data from the end‐expiration (“Expiration”) and end‐inspiration (“Inspiration”) phases of the respiratory cycle. We found that there was not a statistically significant difference in the number of diameter measurements (*P* = 0.1192) nor vessels detected (*P* = 0.2690) measured between Expiration and Inspiration (Fig. 3F, G). The median diameter did significantly decrease from 0.20 mm (IQR: 0.15–0.28) to 0.18 mm (IQR: 0.14–0.26) with inspiration (*P* = 0.0426; Fig. 3E, Table 1). The mode for these scans, i.e., the peak number of measurements, occurred at 0.17 mm for both Inspiration and Expiration (Fig. 4B).

We hypothesized that total pulmonary blood volume would increase with inspiration due to lung expansion and concomitant vascular expansion. To test this hypothesis, we measured vascular volumes as described in Methods, and found that pulmonary blood volume did indeed increase with inspiration (*P* = 0.0412; Fig. 3H).

**Figure 5:**
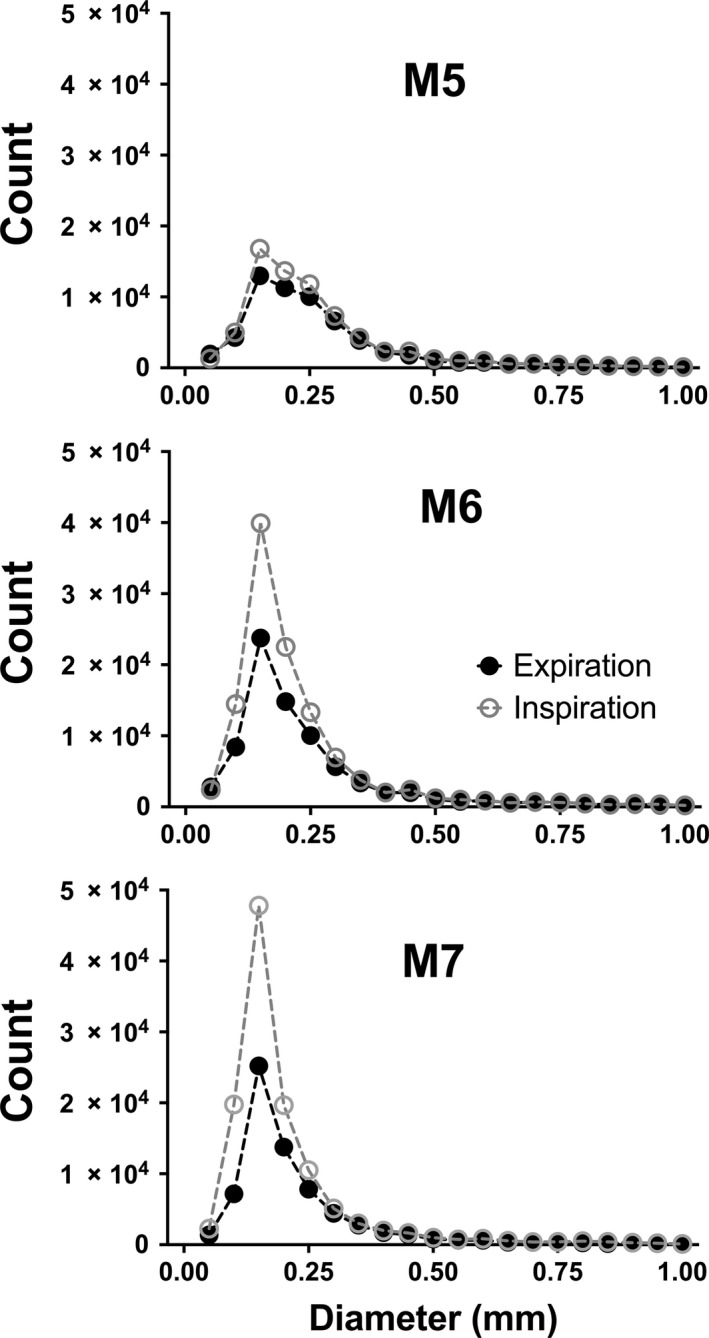
Distribution of diameter measurements from murine pulmonary vascular trees of individual animals from expiration to inspiration during positive pressure ventilation. Histograms of individual data counts are presented as observed numbers per 0.05 mm range of vessel diameters for animals M5‐M7 in the Expiration/Inspiration model.

One mouse (M5) had less variation from Expiration to Inspiration (Fig. 3F–H, Fig. 5), and we wondered if this observation was due to low inspiratory volumes for this animal. To explore this possibility, we compared the relative change in volume over the respiratory cycle for each animal and found that the tidal volumes for M5 (9.2 μL/gm) were similar to those for M6 (9.9 μL/gm) and M7 (8.3 μL/gm). However, we noted that the heart rate for M5 was approximately half, i.e. 138 beats per minute, (bpm) of that in M6 (257 bpm) and M7 (300 bpm) (Videos S1‐S3).

## Discussion

In this study, we applied state‐of‐the‐art lung imaging and image analysis techniques to examine the acute pulmonary vascular responses to hypoxic/CO_2_ gas and positive pressure ventilation in mice. The main finding reported here is that it is now possible to use laboratory‐based x‐ray imaging technology to noninvasively measure dynamic changes over time in the pulmonary vasculature in live mice without intravenous contrast. We were able to characterize and quantify the number and distribution of vessel diameters, and the median and modal diameters before, during, and after exposure to hypoxic/CO_2_ gas. Using this approach, we captured a significant increase in the number of pulmonary vessels detected by the image analysis algorithm 30 min after a brief exposure to hypoxic/CO_2_ gas. In a separate experiment, we characterized the number and distribution of vessel diameters, the relative change in lung volumes, and the absolute change in pulmonary blood volumes over the respiratory cycle during positive pressure ventilation in mice.

Our original goal was to determine whether our in vivo imaging techniques could be used to detect vascular changes reflective of hypoxic pulmonary vasoconstriction (HPV). We performed our 10%O2 + 5%CO2 imaging during a 10 min period of exposure to hypoxic/CO_2_ gas. We used 5% inspired CO_2_ in the hypoxic gas mixture based on literature suggesting that elevated inspired carbon dioxide concentrations augment HPV responses (Noble et al. 1981; Koyama and Horimoto 1982; Orchard et al. 1983; Swenson et al. 1994) and we adapted a protocol used in isolated perfused lungs from BALB/c mice (the strain we used for this study) which included 5% CO_2_ with hypoxic gas challenges to generate increases in pulmonary artery pressures (Weissmann et al. 2004). However, other data suggests that CO_2_ may inhibit HPV (Emery et al. 1977; Chuang et al. 2010), and because we did not directly measure right ventricular pressures during this imaging experiment, we are unable to say with certainty the effect of our ventilatory conditions on HPV.

Indeed, it may not be possible to directly capture HPV using this imaging technique. The absolute cut‐off for resolution of vessels based on the algorithm used in this study is 40–60 *μ*m. That is, no vessels below 40–60 *μ*m can be resolved and measured using this technique. The preacinar vessels that are generally considered to be involved in HPV are in the range of 30–50 *μ*m in mice (Tabuchi et al. 2008) which would be below the limit of resolution, although murine HPV responses have been documented in vessels in the range of 70–100 *μ*m (Paddenberg et al. 2006; Sonobe et al. 2011), and in a wide range of vessel sizes throughout the vascular tree in other species (Hillier et al. 1997; Sylvester et al. 2012). Another challenge to detecting HPV with this imaging approach is that the percent change in vessel diameters directly visualized during HPV may be relatively small at 7% (Tabuchi et al. 2008) to 40% (Paddenberg et al. 2006) and even if the vessels participating in HPV in mice can be visualized, subtle changes in diameter may not be detectable.

The anatomy of the pulmonary vasculature is one in which branching vessels increase in number with increasing generations of branching, and so the drop‐off in the number of vessels detected at diameters below the mode (that is, the diameter at which the peak number of vessels are detected) represents a loss of detected vessels at smaller diameters (under the same imaging and ventilation conditions). This is an inherent limitation of many imaging approaches; there is a point at which the data obtained will be truncated.

The finding of a significant increase in the number of measurements obtained by the algorithm 30 min after exposure to hypoxic/CO_2_ gas was striking and unexpected, and the physiological explanation seems unclear. To understand this finding, we first determined that the number of diameter measurements obtained by the algorithm reflects an increase in the number of vessels *detected*, rather than an increase in the length of existing vessels (which would also lead the algorithm to generate more diameter measurements). Next, we noted that the number of diameter measurements were increased specifically in the smaller diameter range, with the largest number detected at 170 μm. This suggests that vessels that were below the limit of resolution on the baseline and 10%O_2_ + 5%CO_2_ scans dilated into a detectable range on the later scans.

Our second aim in this study was to determine whether we could detect vascular changes over the respiratory cycle with positive pressure ventilation using a noninvasive, noncontrast imaging technique. Others have used in vivo imaging techniques with intravenous contrast to evaluate blood volume changes during mechanical ventilation. Badea et al. (2012) used dual energy micro‐CT imaging to study the volumes of air, lung tissue, and blood in mice during positive‐pressure ventilation with an intravenous liposomal iodine preparation to allow for repeat measurements of blood volumes without the need for repeat iodine injections. These authors were interested in studying the relative distribution of these components with varying amounts of PEEP, and found regional differences in the blood volume variation over the respiratory cycle and in response to PEEP. Porra et al. (2017) also used dual energy x‐ray imaging (which they termed K‐edge subtraction imaging) performed at a synchrotron facility with intravenous iodine as a contrast agent to study regional changes in blood volume in rabbits over the respiratory cycle, again at different PEEP settings. They found that during positive pressure inspiration, regional blood volumes decreased.

Our findings add to these reports. We found a significant increase (*P* = 0.0412) in total pulmonary blood volume with inspiration, while the median vessel diameter decreased from 0.20 to 0.18 mm (*P* = 0.0436). We speculate that the increase in total blood volume with inspiration could reflect an increased blood volume in the conduit vessels. That is, with inspiration during positive pressure ventilation, larger (fewer) vessels may increase slightly in diameter, accounting for the increase in total pulmonary blood volume. The decrease in median vessel diameter may represent an actual decrease in individual vessel diameters. Alternatively, it may be due to the trend (although not statistically significant) toward an increased number of small vessels detected with inspiration, which would also shift the median diameter to the left. As with the Hypoxia/CO_2_ data, this may suggest a dilatation of smaller vessels into the range of detection with inspiration.

This finding might at first glance seem to contradict the findings of Porra et al. However, we measured the pulmonary blood volume of noncapillary vessels only, because alveolar capillaries are below the limit of resolution for our technique. In contrast, Porra et al. specifically excluded larger vessels from their analysis because they were interested in the effects of positive pressure, lung tidal volumes, and PEEP on the capillary blood volume. Therefore, it is likely that we are capturing the increased blood volumes of extra‐alveolar vessels due to radial traction from lung expansion, whereas Porra et al. measured the decreased volumes in alveolar vessels during inspiration related to positive alveolar pressures and compression. Lung blood volume during positive pressure ventilation varies depending on the balance of compression of the alveolar capillaries from intra‐alveolar positive pressure versus increased interstitial radial tethering forces in extra‐alveolar vessels with lung inflation, as well as the pressure gradients between pulmonary arteries and pulmonary veins (Permutt et al. 1961; Graham et al. 1982; Brower et al. 1985; Kuebler 2017). In addition, changes in intrathoracic pressure caused by positive pressure ventilation alter the return of venous blood to the right side of the heart. For an excellent summary of this interesting physiology, we refer readers to Kuebler's commentary on Porra et al.'s work (Kuebler 2017). Determining the relative contributions of these various factors in our model was beyond the scope of this report. It is interesting to note that one of the mice (M5) had a heart rate that was half of the rate for M6 and M7 (as shown in Videos S1‐S3) and that this animal had less variation in blood volume and other measures from expiration to inspiration.

There are several limitations to our data. First, we did not distinguish between pulmonary arteries and pulmonary veins for this analysis. Second, this technique provides robust data on vascular diameters, with 30,000 to over 100,000 measurements per vascular tree, but at the present time we are only able to report a distribution of data points rather than tracking changes in individual vessels. Finally, vascular diameters and pulmonary blood volumes do not reflect pulmonary blood flow, which is dependent on both cardiac output and pulmonary vascular resistance.

In summary, we report the application of a new in vivo imaging approach using advanced x‐ray based imaging technology in conjunction with post‐image acquisition analysis to generate three‐dimensional pulmonary vascular trees from mice. This analysis provides detailed quantitative information about the number and diameter distributions of pulmonary vessels without the need for intravenous contrast. We applied this approach to measure the murine pulmonary vascular response during and after exposure to hypoxic/hypercarbic gas, and to characterize the pulmonary vascular bed over the respiratory cycle during positive pressure ventilation. An important read‐out of this approach may be the change in the number of vessels detected as a reflection of small vessel dilatation into the limits of resolution. This noninvasive imaging modality provides information on the entire vascular bed and can be repeated in the same animal over time, adding a complementary approach to other in vivo and ex vivo methodologies to study the pulmonary vasculature in small animal models.

## Conflict of Interest

AF is CEO of 4Dx. AF, RC, HJ, and SD have stock ownership in 4Dx. AF holds a patent relating to CFPA, and HJ and AF hold a joint patent related to CFPA.

## Supporting information




**Videos S1‐S3**: Real‐time imaging of mouse thorax during CT scan acquisition. Mice M5 through M7 are shown here on Videos S1‐S3, respectively, in a real‐time image that is visible in the control room while the 4DCT scan is acquired.
